# Adenosine as an Active Ingredient in Topical Preparations Against Hair Loss: A Systematic Review and Meta-Analysis of Published Clinical Trials

**DOI:** 10.3390/biom15081093

**Published:** 2025-07-28

**Authors:** Ewelina Szendzielorz, Radoslaw Spiewak

**Affiliations:** Department of Experimental Dermatology and Cosmetology, Faculty of Pharmacy, Jagiellonian University Medical College, ul. Medyczna 9, 30-688 Krakow, Poland; ewelina.szendzielorz@uj.edu.pl

**Keywords:** adenosine, clinical trials, systematic review, meta-analysis, hair loss, androgenetic alopecia, telogen effluvium, female pattern hair loss, baldness, topical treatment

## Abstract

Research results suggest the potential of topical adenosine as a hair-promoting agent. The aim of this study was to examine the available clinical evidence of the efficacy of topical adenosine products in hair loss. This systematic review was conducted in accordance with PRISMA and PICO guidelines and included articles indexed in PubMed, Scopus, and Web of Science. The strength of evidence was assessed according to the GRADE system. Wherever feasible, data were extracted for a meta-analysis. Among 8625 articles returned by the query, 7 clinical trials were identified of topical adenosine (lotion, shampoo) in hair loss. They unanimously reported on a reduction in hair loss and increase in hair density (strength of evidence very low to moderate). A meta-analysis of three eligible trials showed a tendency to increased hair density (OR = 1.03, 95% CI: 0.89–1.20, *p* = 0.68), an increase in thick hair (OR = 1.4, 95% CI: 0.82–2.38, *p* = 0.21) and a decrease in thin hairs (OR = 0.93, 95% CI: 0.61–1.43, *p* = 0.75) after 6 months of alopecia treatment with a 0.75% adenosine lotion. The results from clinical trials published until now suggest that topical adenosine increases hair thickness, reduces excessive hair loss, stimulates hair regrowth, and increases hair density. The overall strength of evidence remains low due to flawed design and small sample sizes in most trials. Nevertheless, topical adenosine products seem worth trying, especially in the case of contraindications or adverse effects to approved medicinal products for hair loss. Further, better designed trials of adenosine in hair loss are warranted.

## 1. Introduction

Adenosine is a nucleoside consisting of adenine and D-ribose [1]. In addition to being components of RNA and DNA, adenosine and its derivatives play key roles in biological processes including energy transfer by adenosine triphosphate (ATP) and adenosine diphosphate (ADP), as well as signal transduction by cyclic adenosine monophosphate (cAMP). Adenosine itself is involved in virtually every aspect of cellular function as an endogenous cell signaling and modulating agent, neurotransmitter, and potent vasodilator [2,3,4]. These functions are fulfilled by the up or downregulation of cAMP, inositol triphosphate (IP3), and IP3/diacylglycerol via G-protein-coupled adenosine receptors divided into four types: A_1_, A_2A_, A_2B_, and A_3_ [5]. Adenosine is produced by chemical synthesis, degradation of RNA, or microbial fermentation using *Bacillus subtilis* [6]. Since adenosine occurs in virtually all living organisms, it is considered a safe substance. Therefore, the US Food and Drug Administration (FDA) and the European Medicines Agency (EMA) have approved topical adenosine preparations for skin application at concentrations of up to 0.1% [7].

Excessive hair loss may be a consequence of systemic diseases, medications, as well as micro- and macronutrient deficiencies [8,9,10,11]. Although baldness is not a life-threatening condition, it causes a great deal of psychological burden [12]. People affected by this problem are more likely to suffer from social phobia, anxiety, and depression [13]. Due to the growing numbers of people suffering from excessive hair loss and alopecia, there is a constant demand for new therapeutic methods [14]. At present, there are only two FDA and EMA-approved therapeutics for the treatment of baldness and excessive hair loss: topical minoxidil and oral finasteride [15]. The treatment of hair loss, however, should be tailored to the actual etiology. Topical therapies seem safer because of the reduced risk of systemic side effects, but the efficacy of topical therapeutics depends on their effective delivery to target structures [16]. Skin penetration by active molecules has been the subject of research in the pharmaceutical industry for many years. However, this is mainly in the context of transdermal delivery of drugs intended to act systemically. The skin, as the outermost and largest organ of the human body with its surface area of about 1.8 m^2^, is a very attractive route for drug administration. Nevertheless, its primary role as a barrier organ and its unique structure developed to fulfill this task considerably limits the entry of xenobiotics. The passage through the skin barrier depends on the molecule’s thermodynamics in the donor phase and modifications to the barrier function by the vehicle [17]. Hair follicles and sweat glands may serve as alternative “bypass” routes for some active substances [18]. Lipophilic carriers can improve the penetration of molecules into hair follicles; however, the penetration rate depends on the momentary activity of the hair follicle [19]. It seems that hair follicles are more “open” in the active growth phase, because the growing hair pushes out sebum plugs that block the follicle openings [20]. Below the level of the sebaceous gland outlets in the hair follicle, the stratum corneum is immature and incapable of effective barrier function. This allows for a rapid penetration of xenobiotics from the follicle into the surrounding network of capillaries and further into the bloodstream [21]. In the context of hair loss treatment, accumulation and retention of an active molecule in the hair follicle seems more desired than its rapid passage into circulation. Indeed, it seems that hair follicles may serve as efficient reservoirs, which could allow for a less frequent application of active molecules [22].

**Figure 1:**
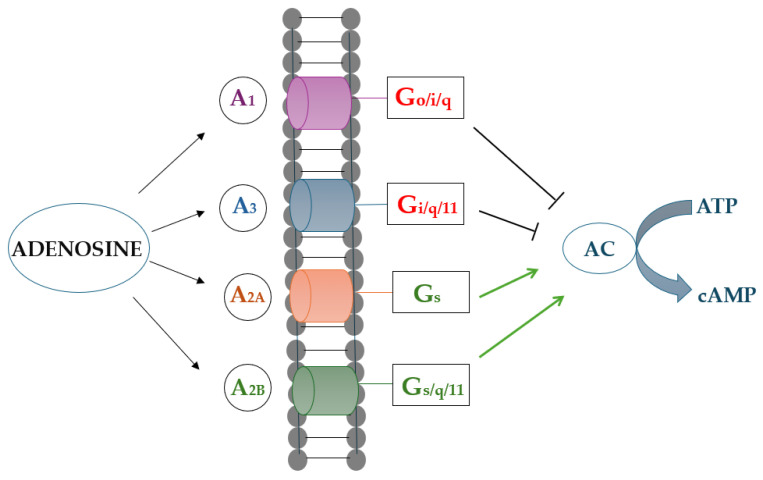
Adenosine receptor signaling: Extracellular adenosine activates four subtypes of receptors: A_1_, A_2A_, A_2B_, and A_3_, which are directly coupled to the G-protein. Depending on the prevailing type of activated receptors, upregulation or downregulation of adenylate cyclase (AC) occurs, an enzyme that converts adenosine triphosphate (ATP) to cyclic adenosine monophosphate (cAMP).

Among potential therapeutic agents for excessive hair loss, adenosine stands out as a strong regulator of hair growth. It is also capable of penetrating into human skin, and the penetration rate can be modulated by adjusting composition of the vehicle [17]. Adenosine acts via G-protein-coupled adenosine receptors located on the cell membrane [23]. It binds to the A_2B_ receptor on dermal papilla cells, leading to an increase in cAMP levels, FGF-7 expression, and activation of the Wnt/β-catenin pathway leading to an increased expression of genes governing cell proliferation in the hair follicle [24,25]. A_2B_ receptors are most abundant on keratinocytes in the outer sheath of the hair root [26]. Adenosine stimulates the proliferation of human hair dermal papilla cells (DPCs) and increases the rate of epithelial cell differentiation in the hair bulb, which is crucial for the induction and maintenance of hair growth [27,28,29]. In cultured DPCs from human scalp hair follicles, upregulation of adenosine A_2B_ receptors increases fibroblast growth factor 7 (FGF-7) and FGF-2 gene expression up to twofold and increases intracellular levels of cAMP in a dose-dependent manner [24]. FGF-7 induces and maintains the anagen (growth) phase in hair follicles [30]. On the cellular level, adenosine drives hair follicles into the anagen phase and prolongs its duration, thereby increasing hair growth [14]. The extension of anagen and subsequent reduction in the telogen phase manifests itself by increased hair density. The therapeutic effects of minoxidil against hair loss are actually mediated by adenosine via A_1_ and A_2_ receptors [27]. These observations altogether suggest that adenosine may be a promising intervention targeting various hair growth disorders (Figure 1).

Although the efficacy of the two FDA and EMA-approved drugs, minoxidil and finasteride, is confirmed in many clinical trials and they are available worldwide, many patients are concerned about possible adverse effects [31,32,33,34,35]. Therefore, many patients opt for heavily advertised “natural” or “trichological” shampoos or lotions for scalp application. Virtually all such products are advertised as containing certain “active ingredients”, though in most cases their alleged activity is not supported by any scientific evidence. An analysis of 92 “hair loss products” (shampoos, lotions, conditioners, and serums) sold in Poland in 2018–2019 showed that, among 448 unique substances on the products’ ingredient lists, as many as 207 were advertised as “active”. However, any scientific evidence was only available for eight (4%) of them [36]. A recent study of anti-hair loss shampoos on the Polish market from 2022 to 2024 identified 39 “trichological shampoos” with 112 ingredients advertised as “active against hair loss”. Scientific evidence of any beneficial effects to the hair (at least one human, animal, or laboratory study) was available for 16 (14%) ingredients [37]. Adenosine was not present in any of the products in the first study and in three (7%) shampoos in the latter. The rare presence of adenosine in hair loss products surprised us to some extent because the molecule seemed a good candidate for hair loss products with relatively many studies indicating its efficacy against hair loss. With mechanistic data altogether suggesting the beneficial effects of adenosine on hair, there seems to be a gap in data from clinical trials to verify this in clinical settings. Clinical data seem scarce, dispersed, and of limited quality in terms of study design and strength of evidence. In further explorations of this problem, we undertook the present systematic review to identify and collate all available evidence from clinical trials of topical adenosine in hair loss.

## 2. Materials and Methods

### 2.1. Data Acquisition

This systematic review was performed following the PRISMA and PICO protocols. The study and its protocol were registered in the international systematic review registry PROSPERO (University of York, UK; registration number 2025 CRD420251079450). Relevant articles with results of clinical trials on topical adenosine in hair loss were searched for in the PubMed, Scopus, and Web of Science databases from database inception till the time of this study. Unpublished data or the gray literature were not included in this review.

### 2.2. Search Strategy

Between January and June 2025, scientific publications indexed in PubMed, Scopus, and Web of Science were searched using the following query: (“adenosine” OR “adenine riboside”) AND (hair OR alopecia OR effluvium OR bald OR baldness OR pilo* OR pili). No filters or date limits were applied. The articles returned by the query were analyzed by title and abstract to remove publications unrelated to the topic and duplicates. Articles that passed the initial screening were subjected to a second full-text review by both authors to identify articles that met the PICO criteria listed in Table 1.

### 2.3. Inclusion and Exclusion Criteria

**Figure 2:**
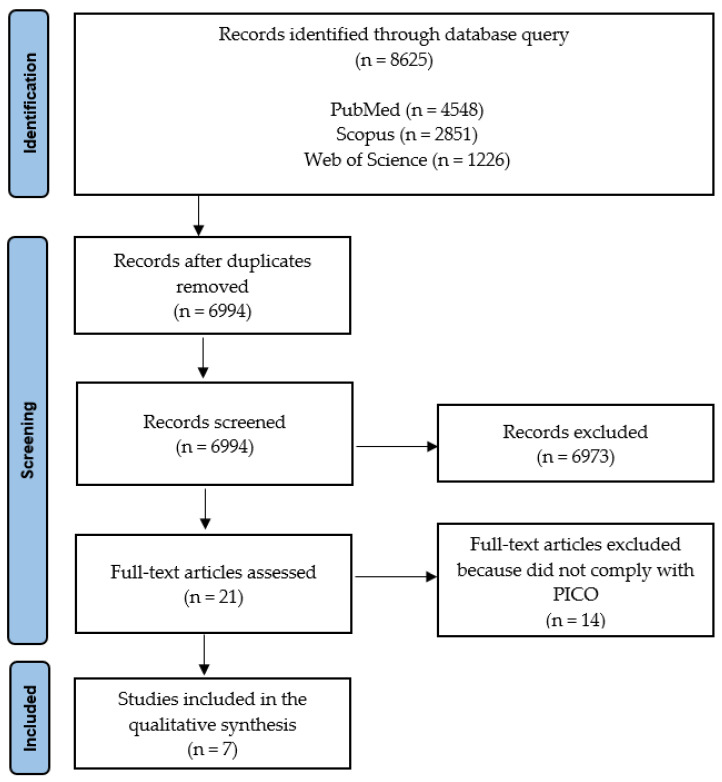
PRISMA protocol for data acquisition.

Only original articles describing results of clinical trials of topical preparations containing adenosine in patients with hair loss were included in the analysis. Review articles, animal and in vitro studies, as well as original research in humans published only as abstracts, posters, or meeting reports, were not included. Priority was given to randomized controlled trials (RCTs) of adenosine-containing topical preparations with well-defined comparators to ensure the strength of evidence. Special attention was paid to adenosine concentration, mode of administration, duration of the treatment, and methods of measuring study outcomes. The entire search and selection process is presented in Figure 2.

### 2.4. Meta-Analysis of Eligible Data

While analyzing the results from the trials included in this systematic review, we sought papers that would report outcomes eligible for pooling and meta-analysis. The criteria for inclusion in this part were (1) the same concentration of adenosine, (2) the same route of administration, (3) the same application and observation period, (4) placebo-controlled study design, and (5) outcome measures that could be expressed in a common unit. For such outcome measures, the meta-analysis with forest plots was carried out with Statistica version 13.3 (Cloud Software Group, Inc., Fort Lauderdale, FL, USA). Data from at least three independent trials are required to carry out the meta-analysis.

## 3. Results

### 3.1. Systematic Review of Clinical Trials

Between 10 January and 30 June 2025, we identified 8625 articles on adenosine. After eliminating 1631 duplicates and 6987 articles that did not meet the inclusion criteria, we ultimately included 7 articles in the final analyses (Figure 2). Four of them presented results from clinical trials of topical products with adenosine as the sole active ingredient. The remaining three trials were of complex topical products with adenosine combined with other substances considered as active. The disclosed concentrations in tested preparations were in the range from 0.2 to 0.75%. In six trials the products were administered to the scalp as leave-on lotions and in one as a rinse-off shampoo. The combined number of subjects participating in all studies was 466, including 239 men with androgenetic alopecia (AGA, 51.3%), 57 women with AGA (12.2%), 30 women with female pattern hair loss (FPHL, 6.5%), and 140 participants (30.0%) in mixed-gender groups with AGA or telogen effluvium (TE), as collated in Table 2.

The participants’ ages in the trials ranged from 18 to 60 years. The observation periods in the studies lasted from 3 to 12 months. Three studies comprised a placebo in the form of identical products without the active substance [38,41,46]. Topical minoxidil 5% was used as a comparator in two studies [39,42], and 0.1% niacinamide in one [44], while there was no comparator in the remaining one [38]. Among studies included in the final review, two [44,46] were assessed as providing moderate strength of evidence according to GRADE, four [38,39,41,42] as low, and one [40] as very low. Factors decreasing the strength of evidence included a lack of placebo, using more than one active ingredient in the formula, which made it impossible to single out the efficacy of adenosine as such, a lack of investigator blinding, the use of arbitrary or vague outcome measures, and a single-center study design. The main results of the trials included in the present review are presented in Table 3 and in more detail in the Supplementary Material (Table S1).

### 3.2. Meta-Analysis

**Figure 3:**
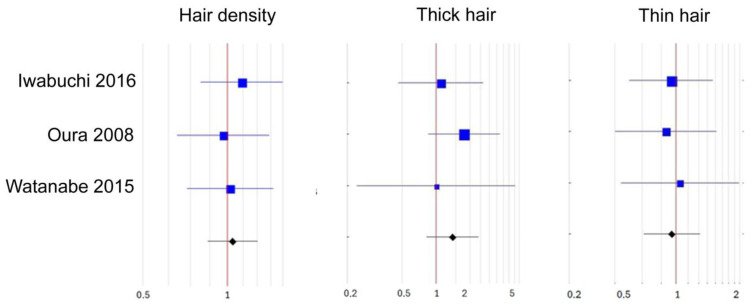
Forest plots depicting meta-analyses of eligible data from clinical trials of adenosine 0.75% lotion versus placebo in androgenetic alopecia (AGA) after 6 months of treatment. Hair density expressed as the number of hairs per 1 cm^2^. Thick hair as the percentage of thick hair (hair diameter > 60 μm). Thin hair as the percentage of thin hair (hair diameter 40–60 μm). Shift from the reference line (red) toward the right favors adenosine; shift toward the left favors placebo. Cumulative odds ratios (ORs), 95% confidence intervals (95% CIs), and significance levels (*p*) for hair density: OR = 1.03, 95% CI: 0.89–1.20, *p* = 0.68; thick hair: OR = 1.4, 95% CI: 0.82–2.38, *p* = 0.21; and thin hair: OR = 0.93, 95% CI: 0.61–1.43, *p* = 0.75, respectively [41,44,46].

We found three studies that were eligible for meta-analysis [41,44,46]. In all, lotions with 0.75% adenosine were used in people with androgenetic alopecia, and the outcome measures shared between them were (1) hair density, (2) percentage of thick hairs, and (3) percentage of thin hairs after 6 months of treatment. In two trials, identical lotions without adenosine were used as a placebo in the control groups [41,46]. In the third study, 0.1% niacinamide lotion was used as the comparator [44]. In an in-depth commentary on this study, Oblong et al. presented evidence that topical niacinamide does not stimulate hair growth [48]. Based on this analysis, we reasoned that 0.1% niacinamide lotion can be regarded as inactive, thus equivalent to a placebo, which, in our opinion, justified the inclusion of this trial into the present meta-analysis. This was necessary because forest plots and meta-analyses require input from at least three separate trials. The overall results showed a low-to-moderate tendency to increased hair density and hair thickness and a decreased rate of thin hair after 6 months of adenosine 0.75% treatment in AGA; however, the difference from the placebo was not statistically significant for either variable (Table 4, Figure 3).

### 3.3. Patient Satisfaction and Safety Data

**Figure 4:**
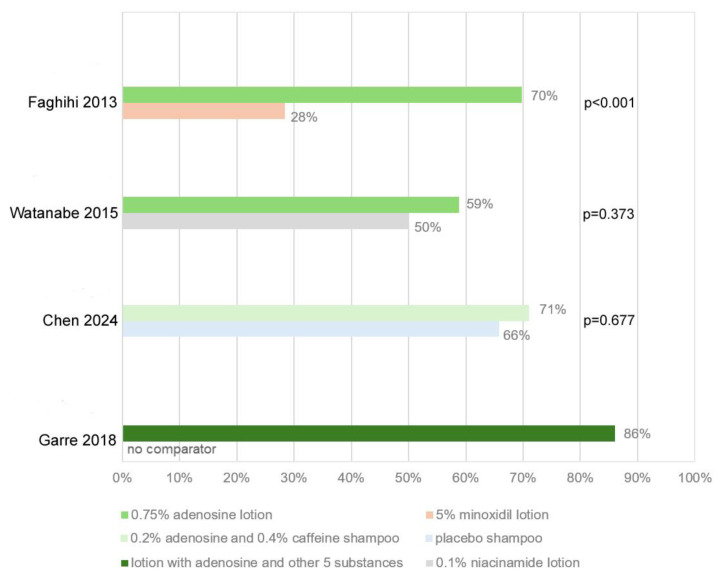
Percentages of patients satisfied with the treatment (adenosine or comparator) with statistical significance of the differences assessed with the chi^2^ test [38,39,40,44].

In one trial, a lotion with 0.75% adenosine was assessed as not inferior to 5% topical minoxidil; however, study participants were significantly more satisfied with the adenosine formula, due to perceived faster effects [39]. In another study of a complex preparation of 0.75% adenosine and two other ingredients, the participants judged that it improved the hair density and thickness slightly better than 5% minoxidil [42]. In an uncontrolled study of a lotion combining adenosine at undisclosed concentration with seven other ingredients, participants were satisfied with the results and declared that they would recommend the product “regardless of the price” [40]. In another study, patients treated with 0.75% adenosine lotion noted better results than the placebo group in terms of hair appearance, hair growth, and hair loss prevention [46]. A “very marked improvement,” “marked improvement,” or “fairly marked improvement” was reportedly observed by 80% of participants treated with a 0.75% adenosine lotion as compared with 32% among those treated with 0.1% niacinamide [44]. Patient satisfaction data, expressed as the percentage of participants satisfied with the treatment, were retrievable from four of the seven trials, including three comparing a product with adenosine versus a comparator. A marked and highly significant difference in favor of 0.75% adenosine lotion as compared with 5% minoxidil lotion was reported in one study [39]. In the other two studies, patients were more satisfied with adenosine preparations than the comparator; however, the differences were not statistically significant (Figure 4).

Ease of use and lack of untoward effects determine the attractiveness and user compliance, which in turn influences the effectiveness of a treatment. Ease of use was addressed in two trials. Cosmetic qualities of a lotion with adenosine, oleanolic acid, apigenin, biotinyl tripeptide-1, 2-4-diaminopyrimidine-3-oxide, ginkgo biloba, and biotin were rated as very high by all study participants, who praised it for a non-greasy, non-sticky consistency and a pleasant scent [40]. In contrast, the shampoo with 0.4% caffeine and 0.2% adenosine made some participants complain of dry hair due to its degreasing properties; nevertheless, 73.7% participants declared that they were very satisfied with its formula [38]. Adverse effects were reported in only one study, in which four participants (9.8%) treated with 5% minoxidil solution (comparator group) and two participants (3.8%) treated with 0.75% adenosine solution complained of complications during treatment. In the minoxidil group, one patient reported hair casts, and three others reported increased hair loss. In the adenosine group, one patient complained of folliculitis, and another one complained of pruritus [39]. In both studies, the differences between adenosine groups and comparator groups were statistically non-significant [38,39]. In another study, none of the participants reported any side effects [41]. The remaining trial reports did not address this aspect.

## 4. Discussion

Hair loss is an immanent part of the hair cycle. However, a disturbed balance between hair growth and hair loss may result in effluvium and, in most severe cases, complete baldness [49]. Clinical trials included in the present review studied the efficacy of topical adenosine in the most common forms of alopecia: androgenetic alopecia (AGA), female pattern hair loss (FPHL), and telogen effluvium (TE). AGA is a non-scarring type of baldness that affects both genders [13,50]. The name “androgenetic alopecia” seems to imply that, as androgen-mediated hair loss, it affects primarily men. However, a study conducted by the Health Insurance Review and Assessment Service (HIRA) in 2021 found that, among 242,960 people with AGA, 44% were women [51]. AGA is an autosomal dominant disorder characterized by the gradual transformation of terminal hair into intermediate and vellus hair. This process involves miniaturization of the hair follicle, shortening of the anagen phase, and a prolongation of the telogen phase, which manifests as hair thinning and shortening and eventually balding [52]. The incidence of AGA increases with age to affect approximately 50% women and 80% men at the age of 70 [53]. It is more common in Caucasians than African Americans or Asians [54]. In men, AGA causes visible recession of the frontal-temporal hairline and hair loss on the vertex, while, in women, the frontal hairline is preserved, and hair loss is mainly visible on the vertex [55]. FPHL may also be referred to as “androgenetic alopecia in women” [56]. The mechanisms in FPHL are the same as in AGA, but FPHL is characterized by progressive thinning of hair on the scalp in a diffuse pattern, in contrast to the male pattern of AGA [57]. TE is a type of non-scarring alopecia that occurs when an increased number of hairs prematurely transition from the active growth phase (anagen) to the resting phase (telogen), resulting in decreased hair density, hair thinning, and diffuse shedding [58]. A typical feature of TE is that hair loss on the scalp is most pronounced 2–3 months after a trigger event [59]. Known trigger factors of TE include psychological stress, childbirth, weight loss, systemic infections, or adverse drug reactions [60]. Despite the high incidence of alopecia, therapeutic options are relatively limited compared with other dermatological disorders [43].

In the trials included in the present systematic review, the dominant topical formulation tested was lotion with 0.75% adenosine—it was used in five of the seven trials covered. In one trial, adenosine 0.75% was part of a complex product with panthenol and niacinamide; therefore, it seems impossible to assess how much of the observed effect could be ascribed to adenosine as such [42]. Looking at the four trials of lotions with 0.75% adenosine as the sole active ingredient, one trial demonstrated the non-inferiority of such lotion compared to 5% minoxidil [39]. Three remaining trials showed an increase in hair thickness and density in response to 0.75% adenosine as compared with placebo in two trials [41,46] and 0.1% niacinamide in one [44]. As mentioned above, convincing scientific evidence indicates that niacinamide has no effect on human hair growth; thus, it might be considered as another form of placebo [48]. Finally, a combination of 0.2% adenosine with 0.4% caffeine was used in one study [38]—in this case, the cumulative effect of both active agents could be equivalent to a higher dose of adenosine alone, based on the fact that caffeine stimulates adenosine receptors A_2A_ and A_2B_, leading to an increase in cAMP levels—an effect strikingly similar to that of adenosine itself [61]. This may also explain the generally positive outcomes in clinical trials of topical caffeine in hair loss presented in our previous systematic review [62]. When discussing whether to choose adenosine or caffeine as the active ingredient for topical preparations (shampoos, lotions) against hair loss, the final decision may depend on secondary factors like bioavailability, pharmacodynamics in the hair follicle, tolerability, stability in topical products, as well as the price of either ingredient.

The number of trials of topical adenosine against hair loss in humans published to date is rather limited; moreover, they may be subject to a considerable publication bias. Publication bias occurs when the outcome of an experiment or research study influences the decision whether to publish it or not [63]. Hopewell et al. demonstrated that trials with positive findings are published more often and more quickly than trials with negative results [64]. On the other hand, van Lent et al. in their study of leading peer-reviewed medical journals showed that manuscripts reporting positive results were in fact not more likely to be published; nevertheless, they suggested that publication bias may occur prior to submission [65]. Authors may be less inclined to publish negative results, considering them as “less interesting”. They also may consider such results as “less relevant” while confusing statistical significance with clinical or real-life relevance. Publication bias should especially be suspected when available evidence comes from a number of small studies, most of which have been commercially funded [66]. This may also be a cause for concern in the present study: out of seven trials included in this review, a lack of authors’ conflict of interest was declared in two studies [38,46], and four were industry-founded or sponsored [40,41,42,44], while the remaining article contained no statement with this regard [39]. The strength of scientific evidence was deemed as low in most trials due to numerous flaws in study design, e.g., small sample size, using complex formulas that made it impossible to single out the efficacy of adenosine, a lack of investigator blinding or placebo, or the use of arbitrary or vague outcome measures. Unfortunately, none of the reports in the present systematic review included any discussion by the authors themselves of a possible bias, confounding factors, or other study limitations.

Well-designed studies are aimed at minimizing the risk of “intrinsic” bias by applying adequate sizes of study groups, randomization, and blinding. Without all these safeguards in place, a study is burdened with a higher risk of bias [66]. Next to the blinding of both participants and investigators, another important means of limiting the observation bias is the selection of an appropriate comparator. Preferably, this should be an identical-looking and smelling product with all the ingredients present in verum, except the active substance of interest. Blinding and proper placebo are pivotal in trials of hair loss in humans, where seasonal fluctuations in spontaneous hair shedding might be a significant confounder [62]. Of the seven trials included in the present systematic review, a placebo was applied in only three [38,41,46]. Another factor limiting the credibility of final conclusions is the lack of dose–response studies, as discussed in detail elsewhere [62]. As mentioned in the introduction, the FDA and EMA approved the use of adenosine at a concentration of 0.1%, while concentrations used in the trials were up to 0.75%. It is thus not clear whether the approved concentration would be efficacious against hair loss. Based on our previous experience, we proposed a set of postulates toward future trials of topical treatments in hair loss [62]. Beyond that, comparability of outcome measures is a most crucial prerequisite to enable the proper estimation of bias (e.g., funnel plot), data pooling, and meta-analyses of results from different studies, as well as credible comparisons of various treatments against hair loss [66]. To achieve this goal, a set of core outcomes measured in a standardized manner in all future trials of hair loss treatments should be elaborated in a consensus of experts in this field. Such sets of core outcomes were already proposed for trials in other dermatological conditions [67,68,69].

When comparing results from trials of topical formulation in hair loss, the differences between various formulations should be kept in mind because they greatly influence the pharmacodynamics of the active agent. For example, lotions are leave-on products with longer-lasting contact allowing for a more thorough penetration into the skin. Six out of seven trials included in the present systematic review tested adenosine in a lotion. Shampoos seem to be the form of topical application that is closest to the daily routine, but, as rinse-off products, they provide shorter contact of the active substance with the scalp after which it is washed off. This may explain why all but one included trials studied lotions, rather than shampoos. The composition of the vehicle may greatly influence the bioavailability and stability of an active agent; therefore, future studies should disclose the composition of used vehicles. The influence of product ingredients and formulations on the penetration into skin and hair follicles was recently discussed elsewhere [61].

## 5. Conclusions

The results from clinical trials published until now suggest that topical adenosine increases hair thickness, reduces excessive hair loss, stimulates hair regrowth, and increases hair density. The overall strength of evidence remains low due to flawed design and small sample sizes in most trials. Nevertheless, topical adenosine products seem worth trying, especially in the case of contraindications or adverse effects to approved medicinal products for hair loss. Further, better-designed trials of adenosine in hair loss are warranted.

## Figures and Tables

**Table 1:** Inclusion criteria for the human studies included in this systematic review.

PICO Criterion	Description
Patients	People suffering from baldness, hair loss, effluvium, or alopecia
Intervention	Adenosine in topical anti-hair loss preparations
Comparator/Control	Placebo or other topical anti-hair loss preparations, lack of controls
Outcomes	Phototrichogram, trichoscopy, investigator assessment, participant assessment

**Table 2:** Types of hair problems covered by clinical trials included in the present systematic review.

Types of Hair Loss	Clinical Trials	Classification and Severity Grading Systems
Androgenetic alopecia (AGA)	[38,39,40,41,42]	The Hamilton–Norwood classification is the most widely used classification system for AGA in men, which has seven stages of disease severity observed from the forehead line to the vertex [43]
	[44]	Ogata created a scale to assess androgenic alopecia dedicated to Japanese men, suggesting that their balding patterns differ from white men. This scale is characterized by the occurrence of six subtypes of balding, each with 2–4 advancement stages [45]
Female Pattern Hair Loss (FPHL)	[38,40,42,46]	The Ludwig classification assesses the degree of baldness in women and categorizes FPHL cases into three grades based on the extent of hair thinning on the top of the head [43]
Telogen effluvium (TE)	[40]	Headington made an attempt at TE classification, but it has not found a broad use, possibly due to its complexity. TE is classified according to the time and cause of the hair transitioning to the telogen phase and is divided into five subtypes, depending on whether the anagen or telogen phase is altered [47]

**Table 3:** Clinical trials of topical adenosine in hair loss.

Year	Study Design	Study Group	Adenosine Formulation	Comparator	Main Outcome	Evidence Strength (GRADE)	Ref.
2008	Randomized double-blind, placebo-controlled trial	30 F with FPHL	0.75% adenosine lotion	Placebo lotion	Objective and subjective improvement	Moderate	[46]
2013	Prospective-randomized study	110 M with AGA	0.75% adenosine lotion	5% MNX lotion	Effects of 0.75% adenosine comparable to 5% MNX	Low	[39]
2015	Randomized double-blind study	102 M with AGA	0.75% adenosine lotion	0.1% niacinamide lotion	Objective and subjective improvement	Moderate	[44]
2016	Prospective randomized study	38 M with AGA	0.75% adenosine lotion	Placebo lotion	Improvement of hair density	Low	[41]
2018	Open-label prospective clinical study	56 M and F with AGA and TE	Lotion with adenosine and oleanolic acid, apigenin, biotinyl tripeptide-1,2-4-diamino pyrimidine-3-oxide, Ginkgo biloba, and biotin	None	Improvement of hair parameters in whole scalp	Very low	[40]
2024	Randomized, controlled, single-blind study	84 M and F with AGA	Shampoo with 0.2% adenosine and 0.4% caffeine	Placebo shampoo	Improvement in hair growth, decrease in hair loss	Low	[38]
2024	Randomized, controlled, double-blind study	46 M and F with AGA	Complex lotion with 0.75% adenosine, 1% panthenol, and 2% niacinamide	5% MNX lotion	Improvement of hair growth, condition, and hair thickness	Low	[42]

Abbreviations: F—female(s); M—male(s); FPHL—female pattern hair loss; AGA—androgenetic alopecia; TE—telogen effluvium; and MNX—minoxidil.

**Table 4:** Hair density per cm^2^ in the studies included in the meta-analysis.

Author, Year	Adenosine Group Before Treatment	Adenosine Group After 6 Months	Placebo * Group Before Treatment	Placebo * Group After 6 Months
Iwabuchi 2016 [41]	243.1	255.0	256.6	246.6
Oura 2008 [46]	210	205	195	195
Watanabe * 2015 [44]	220.2	226.1	230.1	232.7

* A lotion with 0.01% niacinamide was used as the comparator in this study, which we considered equivalent to the placebo, as explained in this text.

## Data Availability

Not applicable.

## Supplementary Materials

The following supporting information can be downloaded at https://www.mdpi.com/article/10.3390/biom15081093/s1, Table S1: Details of clinical studies on the efficacy of topical preparations containing adenosine in the treatment of hair loss [38,39,40,41,42,44,46].

## Abbreviations

The following abbreviations are used in this manuscript:

AGA	Androgenetic alopecia
FPHL	Female pattern hair loss
MNX	Minoxidil
TE	Telogen effluvium
