# Supplementary material for: Adenosine as an Active Ingredient in Topical Preparations Against Hair Loss: A Systematic Review and Meta-Analysis of Published Clinical Trials
Source: Biomolecules. 2025 Jul 28;15(8):1093. doi: 10.3390/biom15081093 (PMC12383921; doi:10.3390/biom15081093)
Supplement: Supplementary file 1 [file biomolecules-15-01093-s001.zip › biomolecules-3690454-supplementary.pdf]

Adenosine as an active ingredient in topical preparations against hair loss: A systematic review and meta-analysis of published clinical trials - Supplementary material

Table S1. Details of clinical studies on the efficacy of topical preparations containing adenosine in the treatment of hair loss.

Name of substances	Study design	Preparation and application	Comparator preparation and application	Inclusion criteria	Number of patients, gender	Duration of treatment	Duration of follow-up	Outcome measures	Main reported outcomes	Authors’ conclusion in the article	Reviewers’ summary	Remarks/ Factors influencing level of evidence	Evidence strength (GRADE))	Ref.
**Adenosine**<br><br>**Caffeine**	Randomized, controlled, single-blind study	I: Shampoo with 0.4% **caffeine** and 0.2% **adenosine**. 10 g of product applied three times a week (rinse-off)	C: Same shampoo without caffeine and adenosine. Same mode of application.	Healthy F and M with self-perceived "thinning hair", Ludwig grade I-1, I-2 or I-3 (F), Norwood Type 2, 2A, 3 or 3A (M), age >18 years.	84 P aged 20-60 , 77 P compl. the study	3 mo	3 mo	Hair density photographs, dermoscopic hair analysis system, hair loss count at combing, hair diameter micrometry, satisfaction scores on PA (quest.)	Mean hair density in I group incr. from 118.25 to 130.03 hairs/cm^2 (up 9.96%, p<0.001), "no significant changes" in C group (data not shown). Mean hair loss at combing decr. in I group from 27.19 to 17.53 (down 35.5%, p<0.001), and in C group from 24.97 to 19.94 (down 20.1%, ns)	“The shampoo containing 0.4% caffeine and 0.2% adenosine exhibited efficacy in regard to reducing hair loss and for hair density enhancement after 3 months compared to the baseline.”	Increased hair density and reduced hair loss after shampoo with caffeine and adenosine. No such effect after sham shampoo.	Not clear how much of the effect is attributable to adenosine. Study not blinded on investigators’ side. Single center study.	Low	[1]
**Adenosine**	Prospective-randomized study	I: group II- 55 P used 1 cc of a lotion with 0,75% **adenosine**, applied twice a day- (leave on).	C: group I- 55 P used 1 cc of a lotion with 5% MNX, twice a day	M with AGA, Norwood-Hamilton stage II-V	110 M, aged under 18	6 mo	6 mo	Macro-photography and counting all visible terminal hairs at a 1-cm2	After 3 months of treatment, relative recovery was achieved in 2.4% and 1.9% of P in group 1 and group 2 (p=0.17). During 6 months, the relative recovery rate did not change either within or between the groups (p=0.99) and after 6 months none of the P achieved complete recovery. P satisfaction rate was significantly higher in group 2 (p=0.003).	“Adenosine has no statistically superiority to MNX in the treatment of AGA according to recovery rates. However, the P were significantly more satisfied with adenosine because of faster prevention of hair loss and appearance of the newly grown hairs.”	Treatment with 0.75% adenosine solution gave similar results to treatment with 5% MNX, which is a promising alternative for people who experience side effects associated with the use of MNX.	Method of measuring effects that is not very reliable.	Low	[2]

**Adenosine**<br><br>**Biotin**	Open-Label Prospective Clinical Study	I: lotion with oleanolic acid, apigenin, biotinyl tripeptide-1, 2-4diamino pyrimidine-3-oxide, **adenosine**, Ginkgo biloba, and **biotin** applied once a day before bed - (leave on).	-------------	M and F with AGA and TE	56 M and F, 24 P with TE and 32 with AGA, aged 25-50	6 mo	6 mo	Phototrichogram, PA quest.	In P with AGA plus TE was a statistically significant increase ($p<0.05$) in the number of anagen hairs and the total number of hairs after 90 and 180 days of treatment.	"When extrapolated from the test area to the whole scalp area (taken to be 500 cm2) this increase represented 5598.2 more hairs per patient at the end of the study."	In the self-assessment questionnaire, the product was rated highly in terms of both effectiveness and cosmetic value - 79%-86%. P were satisfied with the results and would recommend the product regardless of price.	No placebo, and method of measuring effects that is not very reliable, more than one active ingredient makes it impossible to assess the effectivenes s of adenosine.	Very low	[3]
**Adenosine**	Prospective-randomized study	I: 0,75% **adenosine** lotion (leave on).	C: lotion without adenosine	M with AGA	38 volunteers with AGA	6 mo	6 mo	Phototrichogram	Hair density in the adenosine group was significantly higher than in the placebo group ($P = 0.0494$)	"The change in thick hair proportion (classified as hair ≥60 lm in diameter) was significantly different between the two groups ($P < 0.0001$). The proportion of thick hair in the adenosine group was significantly higher than in the placebo group at 6 months".	After treatment of adenosine, a significant increase in hair was observed.	Study not blinded on investigator s' side and method of measuring effects that is not very reliable.	Low	[4]
**Adenosine**<br><br>**Panthenol**	In vivo, ex vivo, and Randomize d study in vitro	I: 1 ml adenosine complex (0.75% adenosine, 1% panthenol, and 2% niacinamide, APN), applied once a day (leave on)	C: 1 ml 5% MNX solution, once a day	M and F with AGA	46 volunteers with AGA, 27 M and 19 W	4 mo	4 mo	Phototrichogram	Hair density significantly increased in the MNX (from 168.1 to 176.5 number/cm2) and APN (from 168.1 to 176.5 number/cm2) groups	"Adenosine showed anti-androgenic activity in vitro and ex vivo in the elongated anagen stages."	The adenosine complex enhanced scalp health and was more susceptible to hair thickness growth via anti-androgenic activity.	More than one active ingredient makes it impossible to assess the effectivenes s of adenosine.	Low	[5]

**Adenosine**	Double-blind, randomized ,placebo-controlled trial	I: 3 ml 0,75% lotion with **adenosine** applied twice a day- (leave on).	C: 3 ml lotion without adenosine (placebo) twice a day	F with FPHL over stage 1.5	30 F, aged 22-53 years	12 mo	12 mo	Phototrichogram , Dermatologist assessments, IA and PA.	85% P in the adenosine-treated group were assessed as "improvement" or "slight improvement" while only 36% P in the placebo-treatment were assessed similarly at month 12.	"The adenosine group was significantly greater than the placebo group regarding: the change in appearance of hair at month 12, the change in hair growth at month 6; and prevention of hair loss at months 6 and 12"	Adenosine improved hair loss in Japanese women by stimulating hair growth and by thickening hair shafts.	-----------	Moderate	[6]
**Adenosine**	Double-blind, randomized study	I: 3 ml 0,75% **adenosine** lotion, applied twice a day- (leave on).	C: 3 ml 0,1 niacinamide lotion as placebo, twice a day	M with AGA, Ogata II-IV stage	102 M, aged 30-50	6 mo	6 mo	Digital microscope, global photographs, PA	Improvements in the thick hair ratio (classified as at least 60 μm in diameter and 80 μm in diameter, P = 0.0331 and P = 0.0268, respectively) were significantly augmented for the adenosine-treated group vs. the niacinamide-treated group. Efficacy in hair density No significant differences in hair density were observed between or within the two groups (P = 0.1831, P = 0.0975, and P = 0.5539, respectively)	"Subjects classified in the 'very clear improvement', 'clear improvement' and 'fairly clear improvement' groups represented 80% of the adenosine-treated participants vs. only 32% of the niacinamide-treated participants".	The improvement in hair thickness index was significantly increased in the adenosine-treated group compared with the niacinamide-treated group.	--------------	Moderate	[7]

Abrreviations: AGA – androgenic alopecia; TE – telogen effluvim; FPHL- female pattern hair loss; compl. – completed; conc. - concentration; F – female(s); I – intervention (verum, test treatment); incr. – increase(d); decr. – decrease(d); C – comparator (placebo, sham treatment); P – participant(s); M – male(s); PA – participant's assessment; IA – investigator's assessment; quest. – questionnaire; ns – non significant; nass – no assessment of statistical significance reported; mo – month(s); w- week(s); d- day(s); MNX- minoxidil; nd- no data.

References:

1. Chen, D.; Yu, F.; Wang, C.; Chen, H.; Tan, J.; Shi, Q.; He, X.; Liu, X.; Wang, F.; Zhao, H. Anti-hair loss effect of a shampoo containing caffeine and adenosine. *J. Cosmet. Dermatol*. **2024**, *23*, 2927-2933. https://doi.org/10.1111/jocd.16347.

2. Faghihi, G.; Iraji, F.; Rajaee Harandi, M.; Nilforoushzadeh, M.A.; Askari, G. Comparison of the efficacy of topical minoxidil 5% and adenosine 0.75% solutions on male androgenetic alopecia and measuring patient satisfaction rate. *Acta Dermatovenerol. Croat.* **2013**, *21*, 155-159.
3. Garre, A.; Piquero, J.; Trullas, C.; Martinez, G. Efficacy and Safety of a New Topical Hair Loss-Lotion Containing Oleanolic Acid, Apigenin, Biotinyl Tripeptide-1, Diaminopyrimidine Oxide, Adenosine, Biotin and Ginkgo biloba in Patients with Androgenetic Alopecia and Telogen effluvium: A Six-month Open-Label Prospective Clinical Study. J. Cosmo. Trichol. **2018**, *4*, 1000132. https://doi.org/10.4172/2471-9323.1000132.
4. Iwabuchi, T.; Ideta, R.; Ehama, R. et al. Topical adenosine increases the proportion of thick hair in Caucasian men with androgenetic alopecia. J. Dermatol. **2016**, *43*, 567-570. https://doi.org/10.1111/1346-8138.13159.
5. Kim, J.; Shin, J.y.; Choi, Y.-H.; Joo, J.H.; Kwack, M.H.; Sung, Y.K.; Kang, N.G. Hair Thickness Growth Effect of Adenosine Complex in Male/Female-Patterned Hair Loss via Inhibition of Androgen Receptor Signaling. *Int. J. Mol. Sci.* **2024**, *25*, 6534. https://doi.org/10.3390/ijms25126534.
6. Oura, H.; Iino, M.; Nakazawa, Y. et al. Adenosine increases anagen hair growth and thick hairs in Japanese women with female pattern hair loss: a pilot, double-blind, randomized, placebo-controlled trial. *J. Dermatol.* **2008**, *35*, 763-767. https://doi.org/10.1111/j.1346-8138.2008.00564.x.
7. Watanabe, Y.; Nagashima, T.; Hanzawa, N. et al. Topical adenosine increases thick hair ratio in Japanese men with androgenetic alopecia. *Int. J. Cosmet. Sci*. **2015**, *37*, 579-587. https://doi.org/10.1111/ics.12235.
